# Deposition of exchange-coupled dinickel complexes on gold substrates utilizing ambidentate mercapto-carboxylato ligands

**DOI:** 10.3762/bjnano.8.139

**Published:** 2017-07-05

**Authors:** Martin Börner, Laura Blömer, Marcus Kischel, Peter Richter, Georgeta Salvan, Dietrich R T Zahn, Pablo F Siles, Maria E N Fuentes, Carlos C B Bufon, Daniel Grimm, Oliver G Schmidt, Daniel Breite, Bernd Abel, Berthold Kersting

**Affiliations:** 1 Institut für Anorganische Chemie, Universität Leipzig, Johannisallee 29, 04103 Leipzig, Germany; 2 Semiconductor Physics, Chemnitz University of Technology, D-09107 Chemnitz, Germany; 3 Material Systems for Nanoelectronics, Chemnitz University of Technology, Reichenhainer Str. 70, 09107 Chemnitz, Germany; 4 Institute for Integrative Nanosciences, IFW Dresden, Helmholtz Str. 20, 01069 Dresden, Germany,; 5 Leibniz-Institute of Surface Modification (IOM), Permoser Str. 15, D-04318 Leipzig, Germany

**Keywords:** ambidentate ligands, chemisorption, gold surfaces, macrocyclic complexes, mercapto-alkanecarboxylic acid

## Abstract

The chemisorption of magnetically bistable transition metal complexes on planar surfaces has recently attracted increased scientific interest due to its potential application in various fields, including molecular spintronics. In this work, the synthesis of mixed-ligand complexes of the type [Ni^II^_2_L(L’)](ClO_4_), where L represents a 24-membered macrocyclic hexaazadithiophenolate ligand and L’ is a ω-mercapto-carboxylato ligand (L’ = HS(CH_2_)_5_CO_2_^−^ (**6**), HS(CH_2_)_10_CO_2_^−^ (**7**), or HS(C_6_H_4_)_2_CO_2_^−^ (**8**)), and their ability to adsorb on gold surfaces is reported. Besides elemental analysis, IR spectroscopy, electrospray ionization mass spectrometry (ESIMS), UV–vis spectroscopy, and X-ray crystallography (for **6** and **7**), the compounds were also studied by temperature-dependent magnetic susceptibility measurements (for **7** and **8**) and (broken symmetry) density functional theory (DFT) calculations. An *S* = 2 ground state is demonstrated by temperature-dependent susceptibility and magnetization measurements, achieved by ferromagnetic coupling between the spins of the Ni(II) ions in **7** (*J* = +22.3 cm^−1^) and **8** (*J* = +20.8 cm^−1^; *H* = −2*J*S_1_S_2_). The reactivity of complexes **6**–**8** is reminiscent of that of pure thiolato ligands, which readily chemisorb on Au surfaces as verified by contact angle, atomic force microscopy (AFM) and spectroscopic ellipsometry measurements. The large [Ni_2_L] tail groups, however, prevent the packing and self-assembly of the hydrocarbon chains. The smaller film thickness of **7** is attributed to the specific coordination mode of the coligand. Results of preliminary transport measurements utilizing rolled-up devices are also reported.

## Introduction

The deposition of switchable transition metal complexes on Au surfaces is a topical research area [1–4] due to the many potential applications such as storage of information at the molecular level [5–7] and in the area of molecular spintronice [8–11]. For a review concerning the organization of electronically bistable molecule or molecular switches on surfaces see [4]. The deposition of single molecule magnets (SMMs) has received increased attention [12–14] and several strategies have been designed to deposit these materials as rows [15], thin films [16–19], or multilayers [20–26]. However, the limited thermal and kinetic stability of most SMMs prevents their thermal evaporation [27] and has initiated the search for milder, solution-based methods for surface functionalization. Of these, the formation of self-assembled monolayers of SMMs appears to be an attractive and suitable method [28–36].

**Scheme 1:**
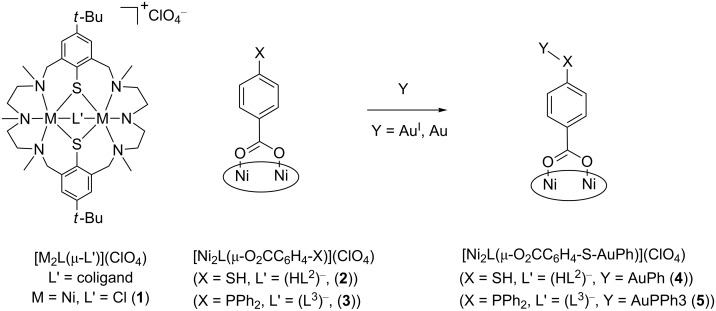
Structure of the dinuclear complexes [M_2_L(μ-L’)](ClO_4_) and representation of the structure of complexes **2**–**5** bearing ambidentate coligands (HL^2^)^−^ and (L^3^)^−^ (the doubly deprotonated macrocycle H_2_L is shown as an ellipse for clarity).

Our work involves the deposition of exchange-coupled complexes of the type [M^II^_2_L(μ-L’)](ClO_4_), where L represents a hexaazadithiophenolate macrocycle (Scheme 1), L’ an ambidentate coligand, and M is a paramagnetic transition metal ion, usually Mn^II^, Fe^II^, Co^II^, or Ni^II^ [37].

**Figure 1:**
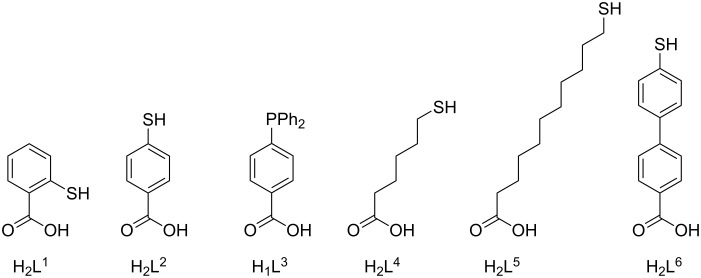
Ambidentate ligands with soft (–SH, –PPh_2_) and hard (–CO_2_H) donor functions.

The ambidentate phosphane-carboxylato [38] and thiol-carboxylato coligands H_2_L^2^ and H_1_L^3^ (Figure 1) were found to bind selectively via their carboxylate function to form the carboxylato-bridged complexes [Ni_2_L(HL^2^)](ClO_4_) (**2**) and [Ni_2_L(L^3^)](ClO_4_) (**3**) [39–40] such that an exposed thiol or phosphane group is available for further functionalization. Indeed, **2** dimerizes via a disulphide bond upon oxidation in air to generate a tetranuclear [{Ni_2_L}_2_(O_2_CC_6_H_4_S)_2_]^2+^ complex, while “auration” of **2** and **3** with Au^I^ sources leads to the trinuclear Ni^II^_2_Au^I^ species **4** and **5**. Moreover, complexes **2** and **3** interact also with Au surfaces via Au–S and Au–P bonds without complex disintegration as established by contact angle, spectroscopic ellipsometry, atomic force microscopy (AFM), X-ray photoelectron spectroscopy (XPS) and scanning tunneling microscopy (STM) measurements [41].

**Scheme 2:**
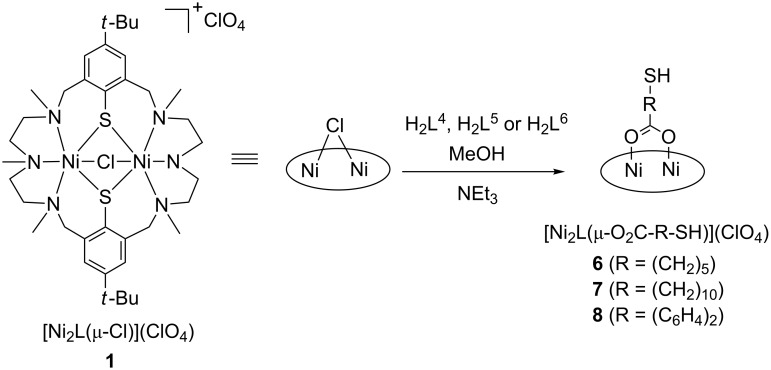
Synthesis of the complexes **6**–**8** (the doubly deprotonated macrocycle H_2_L is represented as an ellipse for clarity).

The present study is an extension of this work and focuses on the synthesis, characterization and deposition of dinuclear [Ni_2_L(L’)](ClO_4_) complexes **6**–**8** bearing the ambidentate coligands H_2_L^4^–H_2_L^6^ (Figure 1, Scheme 2). The crystal structure, reactivity features, and magnetic properties of compounds **6**–**8** are presented along with the results stemming from characterization of the surface assemblies by contact angle measurements, spectroscopic ellipsometry, AFM and transport measurements. To our knowledge, polynuclear macrocyclic complexes have not been anchored to gold via ambidentate mercaptobenzoate ligands. However, polynuclear Mn_12_ complexes have been fixed to gold via perfluorinated mercaptobenzoate linkers [42].

## Results and Discussion

### Synthesis and characterization of complexes

The investigated compounds and their labels are collected in Scheme 2. The compounds were prepared in analogy to the synthesis of [Ni_2_L(HL^2^)](ClO_4_) (**2**) [39]. Thus, treatment of [Ni_2_L(μ-Cl)](ClO_4_) (**1**) [43–44] with a slight excess of the triethylammonium salt of the corresponding mercapto-carboxylate anion (prepared in situ from the free acid and NEt_3_) in methanol at ambient temperature resulted in pale-green solutions. Upon addition of an excess of LiClO_4_ green, air-sensitive compounds of composition [Ni_2_L(L’)](ClO_4_) (where L’ = (HL^4^)^−^ (**6**), (HL^5^)^−^ (**7**), and (HL^6^)^−^ (**8**)) could be obtained in good yields (56–60%). Compounds **6**–**8** are soluble in polar aprotic solvents (e.g., acetone, acetonitrile, or dichloromethane), but are only sparingly soluble in alcohols and insoluble in H_2_O. The products gave satisfactory elemental analyses and the electrospray ionization mass spectrometry (ESIMS) spectra with base peaks for the individual [Ni_2_L(L’)]^+^ cations (Table 1) were consistent with the formulation as mixed ligand [Ni_2_L(L’)](ClO_4_) complexes.

**Table 1:** Selected UV–vis spectroscopy, IR spectroscopy, and ESIMS data and their assignments for compounds **6**–**8**. The data for the reference compounds (**1**–**5**, **9**–**11**) have been included for comparison.

Compound	UV–vis ν_1_, ν_2_ [nm]	IR ν_as_, ν_s_(RCO_2_), ν(SH) [cm^−1^]	ESI [M^+^] *m*/*z*	Ref.

**1** [Ni_2_L(Cl)](ClO_4_)	658, 1002	–, –, –	n.d.^a^	[43]
**2** [Ni_2_L(HL^2^)](ClO_4_)	652, 1125	1599, 1408, 2550	937.2	[39]
**3** [Ni_2_L(HL^3^)](ClO_4_)	650, 1122	1550, 1408, –	1089.4	[40]
**4** [Ni_2_L(L^2^)Au(PPh_3_)](ClO_4_)	653, 1129	1587, 1403, –	1397.4	[39]
**5** [Ni_2_L(L^3^)Au(Ph)](BPh_4_)	651, 1119	1552, 1437, –	1363.3	[39]
**6** [Ni_2_L(HL^4^)](ClO_4_)	649, 1129	1596, 1407, 2547	932.39	this work
**7** [Ni_2_L(HL^5^)](ClO_4_)	650, 1133	1581, 1423, 2547	1002.5	this work
**8** [Ni_2_L(HL^6^)](ClO_4_)	650, 1118	1596, 1408, 2548	1013.37	this work
**9** [Ni_2_L(O_2_CPh)](ClO_4_)	651, 1123	1569, 1407, –	n.d.^a^	[45]
**10** [Ni_2_L(O_2_CCH_3_)](ClO_4_)	650, 1131	1588, 1428, –	n.d.^a^	[45]
**11** [Ni_2_L(SPh)](ClO_4_)	667, 1141	–, –, –	n.d.^a^	[46]

^a^n.d. = not determined.

It has already been demonstrated that the [Ni_2_L]^2+^ dication has a higher affinity for carboxylate ions than for thiophenolate groups, and that the former, when attached to [Ni_2_L]^2+^, invariably act as a μ_1,3_-bridge [45–46]. The UV–vis spectra of **6**–**8** recorded in MeCN solution provided convincing evidence that the present mercapto-carboxylate ligands are also coordinated in this fashion. Similar to **2** and **3**, **6**–**8** exhibit two electronic absorption bands at wavelengths of ≈650 nm and 1120 nm, typical for an octahedral NiN_3_S_2_O carboxylate chromophore (assigned as ν_2_(^3^*A*_2g_→^3^*T*_2g_) and ν_1_(^3^*A*_2g_→^3^*T*_1g_), respectively, in pure *O_h_* symmetry). Infrared spectroscopy (IR) is also a powerful method to examine carboxylate coordination modes [47–48]. As can be seen (Table 1), the present complexes reveal two strong bands, one around 1596–1581 cm^−1^ and the other between 1407 and 1423 cm^−1^, as in other carboxylato-bridged complexes supported by H_2_L [45]. These are assigned to the asymmetric and symmetric RCO_2_^−^ stretching frequencies. Weak bands at ≈2550 cm^−1^ ν(SH) typical for a RSH group are also present.

### Description of the crystal structure of **6** and **7**

**Figure 2:**
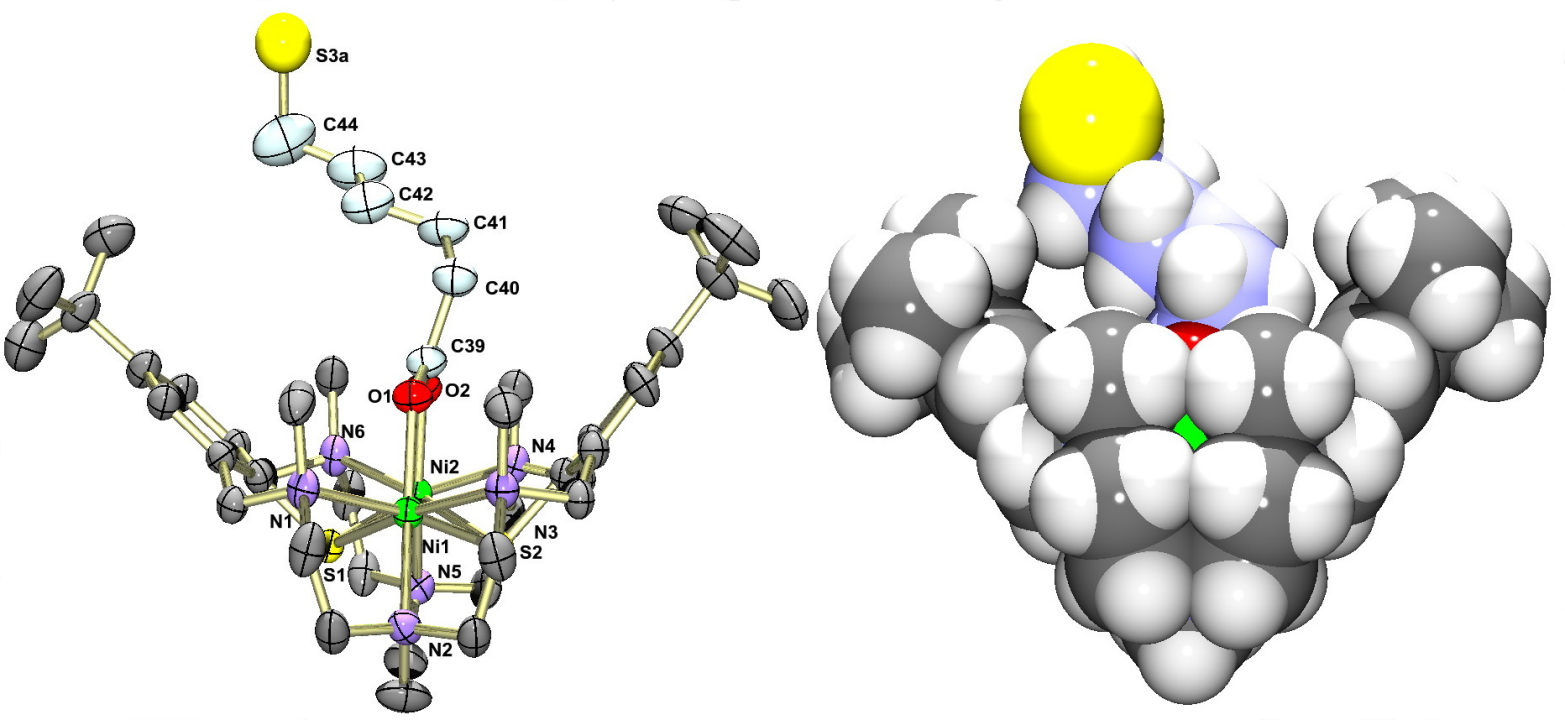
ORTEP (left) and van der Waals representations (right) of the molecular structure of the [Ni_2_L(O_2_C(CH_2_)_5_SH)]^+^ cation in crystals of **6**. The accessibility of the thiol head group (atom labeled S3a) is obvious from this view.

Attempts to grow single crystals of **6**–**8** met with little success. Only preliminary X-ray crystallographic data for the complexes **6** and **7** can be presented. Although the quality of the structure determination is low and insufficient for publication, these data can surely validate the atom connectivity of the [Ni_2_L(L’)]^+^ complex and the binding mode of the coligands. Figure 2 shows ORTEP and van der Waals representations of the structure of the [Ni_2_L(HL^4^)]^+^ cation in **6**, with the mercaptohexanoate unit bridging the two Ni(II) ions in a symmetrical fashion as expected from the spectroscopic data. The [Ni_2_L]^2+^ fragment adopts a cleft-like structure as observed in other carboxylato-bridged complexes supported by L [45]. In contrast to other ω-mercapto-alkanethiols, which adopt an extended zig-zag conformation, the coligand is twisted about the C40–C41 bond (“gauche” conformation) most likely due to steric constraints exerted by the surrounding NMe groups. We have observed similar effects in an azido-bridged complex, where the surrounding alkyl groups dictate the coordination mode of the azido ligand [49].

**Figure 3:**
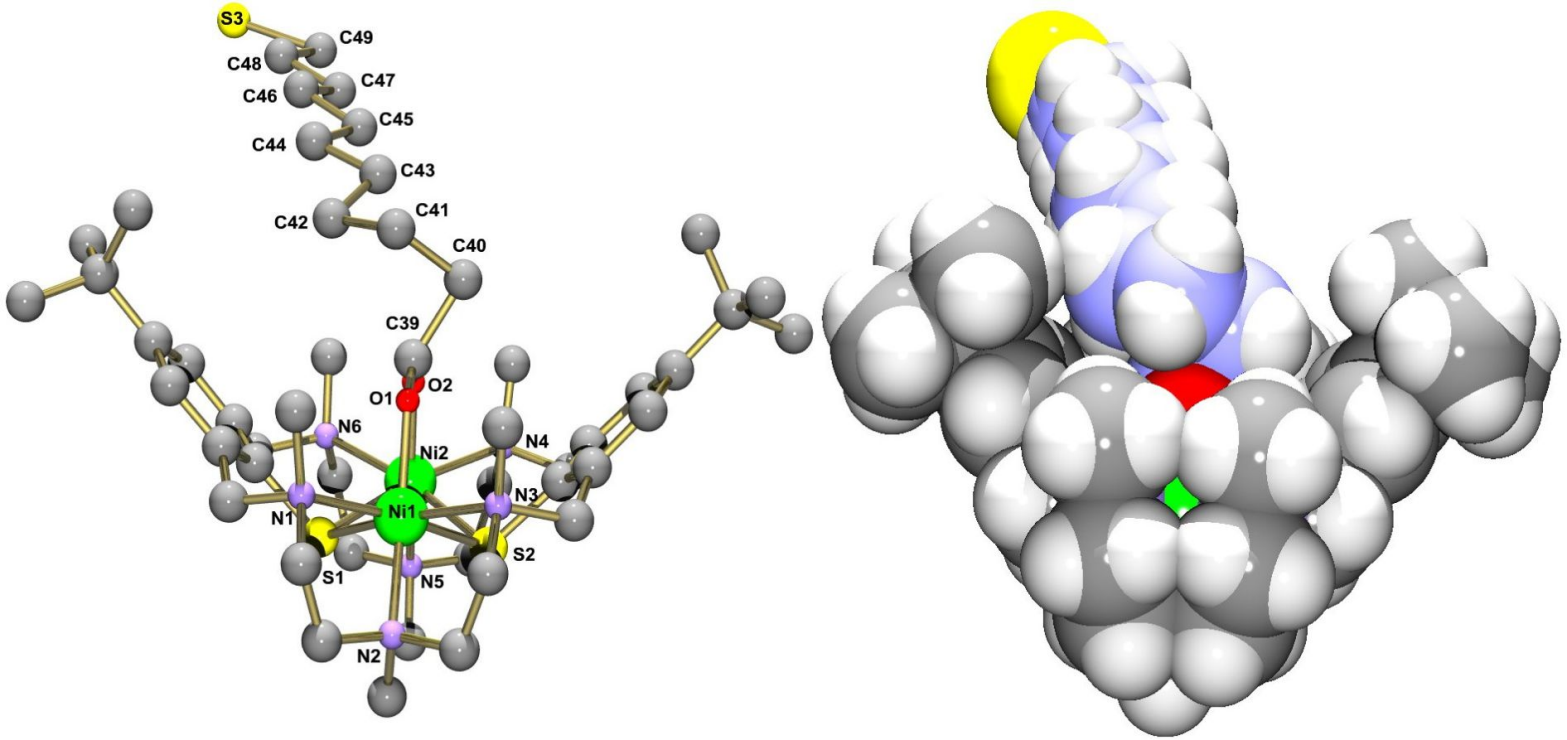
Ball and stick (left) and van der Waals representations (right) of the molecular structure of the [Ni_2_L(O_2_C(CH_2_)_10_SH)]^+^ cation in crystals of **7**.

Figure 3 provides a ball and stick and a van der Waals representation of the molecular structure of the [Ni_2_L(O_2_C(CH_2_)_10_SH)]^+^ cation in **7**. The coligand is again coordinated via its carboxylate function, and the [Ni_2_L]^2+^ fragment is isostructural with that in **6**. Twisting of the coligand is also encountered in this case (about the C40–C41 (β) and C41–C42 (γ) bonds). As in **6**, the undecanoate moiety protrudes laterally out of the binding pocket of the [Ni_2_L]^2+^ fragment. Thus, in all the cases that we have examined so far, coordination of the carboxylato group is strongly preferred over binding through the RSH tail group both in solution as well as in the solid state and there is no ambiguity concerning the regiochemistry of the complexation.

### Magnetic properties of **6** and **7**

The magnetic properties of a series of dinuclear [Ni_2_L(L’)]^+^ complexes with various bridging ligands have been reviewed [37]. All [Ni_2_L(μ-carboxylato)]^+^ complexes are characterized by an *S* = 2 ground state that is attained by a net ferromagnetic exchange interaction with *J* values ranging from approximately +15 to +25 cm^−1^ (H = −2*J*S_1_S_2_) [50].

**Figure 4:**
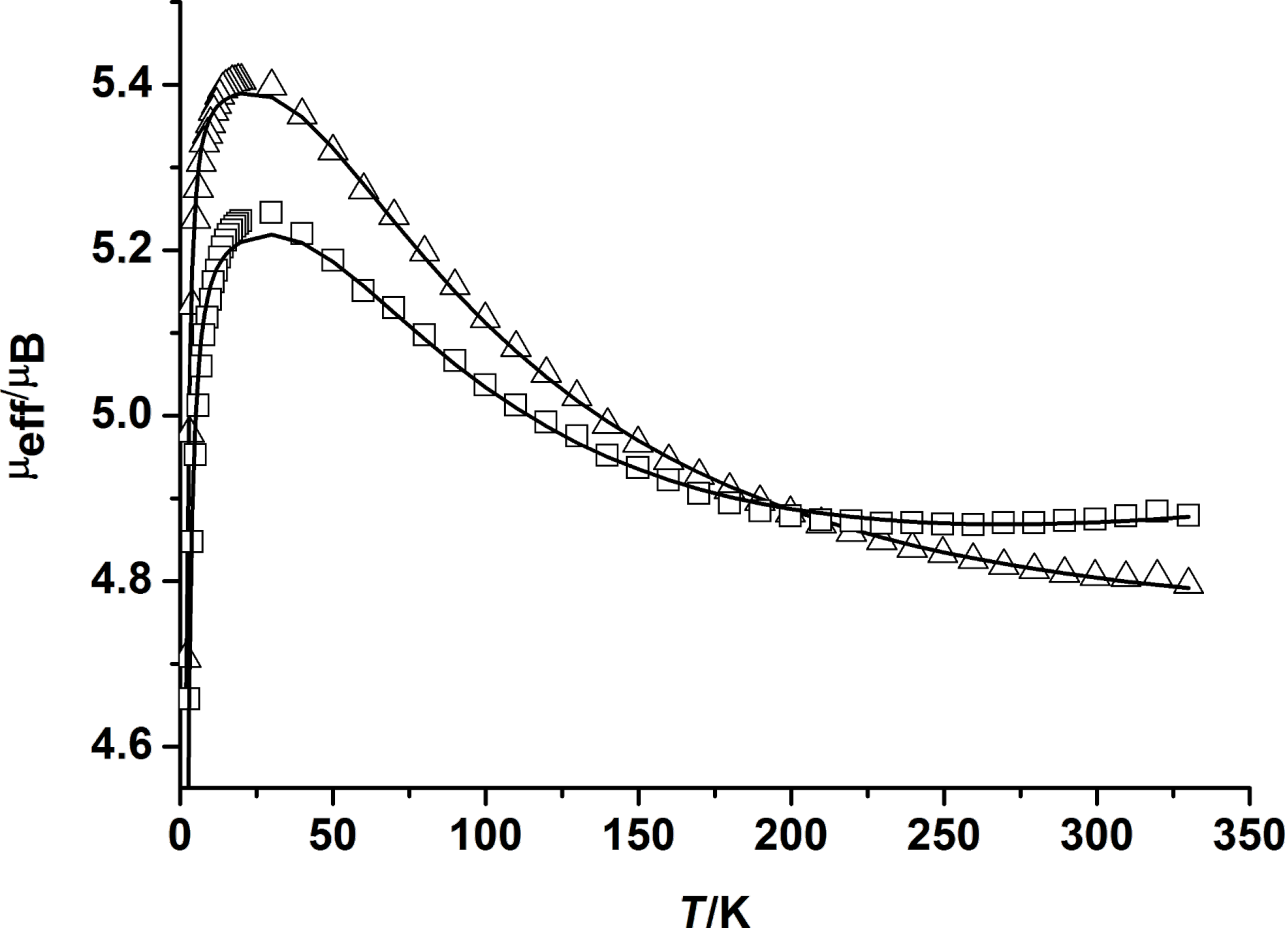
Temperature dependence of the effective magnetic moment μ_eff_ (per dinuclear complex) for **7** (open triangles) and **8** (open squares). The solid lines represent the best fits to Equation 1.

To gain insight into the magnetic properties of the present complexes, variable-temperature magnetic susceptibility data were measured for **7** and **8** between 2 and 330 K in applied external magnetic fields of *B* = 0.1, 0.5, and 1.0 T. Figure 4 shows the susceptibility data (per dinuclear complex) in the form of μ_eff_ versus *T* plots at 1 T. Both complexes behave similarly. Thus, for complex **7**, the effective magnetic moment per dinuclear complex at 300 K increases from 4.78·μ_B_ (**8**: 4.80 μ_B_) at 300 K to a maximum value of 5.36 μ_B_ at 23 K (**8**: 5.27 μ_B_). On lowering the temperature further the magnetic moment decreases to 4.60 μ_B_ (or 4.34 μ_B_) at 2 K. This behavior suggests that the electron spins on the two Ni(II) (*S* = 1) ions are coupled by an intramolecular ferromagnetic exchange interaction. This would lead to an *S*_t_ = 2 ground state, in agreement with other carboxylato-bridged compounds supported by L. The decrease in χ_M_*T* below 20 K can be attributed to zero-field splitting of Ni(II) [51].

The magnetic moment data were analyzed in order to determine the magnetic parameters. The appropriate spin-Hamiltonian (Equation 2) [52] should include additional terms to account for single-ion zero-field splitting for each Ni^2+^ ion. *J* is the exchange coupling constant, *D*_i_, *E*_i_/*D*_i_, and *g*_i_ are the local axial and rhombic zero-field splitting parameters and *g*-values (isotropic average) [53]. It is well-known that temperature-dependent magnetic susceptibility measurements do not allow a concise determination of the magnitude and sign of *D* [54]. As a consequence, the data for **7** and **8** were analyzed with the approximation in Equation 1 [55]. The *D* and *g* values were kept identical for the two nickel atoms.

[2]
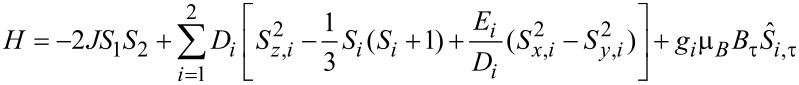


[1]
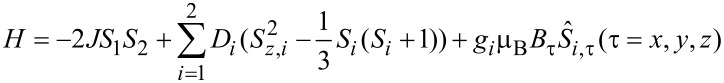


By taking into account the zero-field splitting and temperature-independent paramagnetism (TIP), reasonable fits of the experimental data were possible, yielding *J* = +23 cm^−1^ (*g* = 2.20, *D* = 2.59 cm^−1^) for **7** and *J* = +25 cm^−1^ (*g* = 2.25, *D* = 3.21 cm^−1^) for **8**. The inclusion of the *D*-parameter improved the low-temperature fits significantly, but as stated above, these values by no means represent accurate values [56–57]. The *D*-values obtained from temperature-dependent magnetic susceptibility measurements should be taken as indicative rather than definitive because these measurements are not the most appropriate for the determination of *D*-values. It should also be noted that HFEPR experiments for carboxylato-bridged [Ni_2_L(O_2_CR)]^+^ complexes revealed a negative axial magnetic anisotropy parameter (*D* < 0) with *D*-values of approximately −0.04 cm^−1^, indicative of an easy magnetic anisotropy axis. However, the magnetic anisotropy barrier is too small to allow for sufficient retention of magnetization at finite temperature. The value of *J*, on the other hand, does not significantly depend on *D*. Thus, *J* is unambiguous and provides a correct value for the magnetic coupling in **7** and **8**. The experimental *J*-values agree also reasonably well with those obtained by broken symmetry density functional calculations for exchange interactions (*J* = +26 cm^−1^ for **7**; *J* = +27 cm^−1^ for **8**).

In order to evaluate the coupling through the thiophenolato and carboxylato bridges within the N_3_Ni(μ-SR)_2_(μ_1,3_-O_2_CR)NiN_3_ core, we utilized a breakdown approach in which the carboxylate group was virtually removed to obtain the hypothetical [Ni_2_(L)]^2+^ dication, which was subjected to broken symmetry DFT density functional theory calculations. The details of these investigations will be published elsewhere. This method has previously been shown to be a powerful tool to unravel the contribution of the azido and thiolato-bridges for the complex [Ni_2_L^Me2H4^(μ-N_3_)]ClO_4_ [49], where L^Me2H4^ represents a 28-membered variant of the macrocycle L. The results imply that a moderate "ferromagnetic" contribution of ≈30 cm^−1^ through the μ_1,1_-bridging thiophenolato groups of the supporting macrocycle is counterbalanced by a weak antiferromagnetic interaction (*J* ≈ −5 cm^−1^) through the carboxylato-bridges (*J*_O2CR_), to produce a net ferromagnetic exchange interaction of *J* ≈ 25 cm^−1^. A magneto-structural correlation has recently been reported for related dinuclear nickel complexes of the type [Ni_2_L^Me2H4^(μ-L’)]^+^, where L’ = F^−^, Cl^−^, Br^−^, OH^−^, and N_3_^−^) [58]. The *J*-values were found to depend primarily on the bridging Ni–S–Ni and Ni–L’–Ni angles. The findings made for the carboxylato-bridged compounds are in good agreement with the reported trend.

### Chemisorption of complexes **6–8** on gold surfaces

In view of the results obtained with the complexes **2** and **3**, the deposition of the nickel complexes **6**–**8** on flat gold surfaces was examined. The deposition experiments were carried out in solution according to a protocol developed for the preparation of self-assembled thiol monolayers [59] as the complexes cannot be deposited via the gas phase. They decompose without melting. Thus, clean gold-coated Si wafers were immersed in a 1 × 10^−3^ M solution of the respective complex in MeCN or CH_2_Cl_2_ for 24 h followed by washing with EtOH and drying under N_2_ flow. The modified Au(111) surfaces were examined by contact angle measurements, AFM topography analysis, and spectroscopic ellipsometry. Table 2 lists the results. The data for **2** and **3** and other compounds have been included for comparison.

**Table 2:** Water contact angles, AFM roughness, and optical thickness obtained for Au(111) surfaces modified with various dinickel(II) complexes.

Compound	Contact angle [°]^a,b^	Roughness (rms) [Å]^b,c^	Optical film thickness [Å]	Ref.

bare gold	75.8 (1.5)	6 (1)	–	[39]
**2** [Ni_2_L(L^2^)](ClO_4_)	71.4 (2.1) 75.9 (2.1)^d^ 76.0 (2.0)^e^	17 (5) 16 (2) n.d.^f^	16 (7) n.d.^f^ n.d.^f^	[39], this work
**3** [Ni_2_L(L^3^)](ClO_4_)	71.5 (1.6) 76.7 (1.9)^d^ 76.5 (2.1)^e^	17 (5) n.d.^f^ n.d.^f^	15 (8) n.d.^f^ n.d.^f^	[40]
**6** [Ni_2_L(L^4^)](ClO_4_)	n.d.	n.d.	n.d.	–
**7** [Ni_2_L(L^5^)](ClO_4_)	70.8 (1.0)	13 (4)	20 (7)	this work
**8** [Ni_2_L(L^6^)](ClO_4_)	69.6 (3.3)	19 (4)	24 (7)	this work
**9** [Ni_2_L(O_2_CPh)](ClO_4_)	75.9 (2.0)	6 (1)	n.d.^f^	[40]
**10** [Ni_2_L(O_2_CMe)](ClO_4_)	75.8 (1.5)	6 (1)	n.d.^f^	[40]

^a^The values represent the average of five 4 μL drops of distilled, deionized water. The ″bare″ gold surfaces were identically treated to the modified surfaces except with omission of any adsorbate in the solvent. ^b^Standard deviations are given in parentheses. ^c^Root mean squared (rms) surface roughness determined by AFM. ^d^Tetraphenylborate salt. ^e^After metathesis with NaBPH_4_. ^f^n.d. = not determined.

### Static contact angle measurements, atomic force microscopy and optical thickness

The chemisorption of the perchlorate salts **7** and **8** leads to smaller contact angles (70.8° and 69.6°) than that of bare gold, and the values compare well with those reported previously for **2** and **3** (Table 2). The contact angles are relatively high, particularly considering the ionic nature of the layers, and this may relate to the fact that the charges are well-shielded by the apolar groups of the supporting macrocycle. The contact angles for **2**, **3**, **7**, and **8** should otherwise be compared with those of reference compounds **9** and **10**, which lack end groups for surface fixation (and which are apparently not chemisorbed on the gold surfaces), as suggested by contact angles close to that of the bare gold.

**Figure 5:**
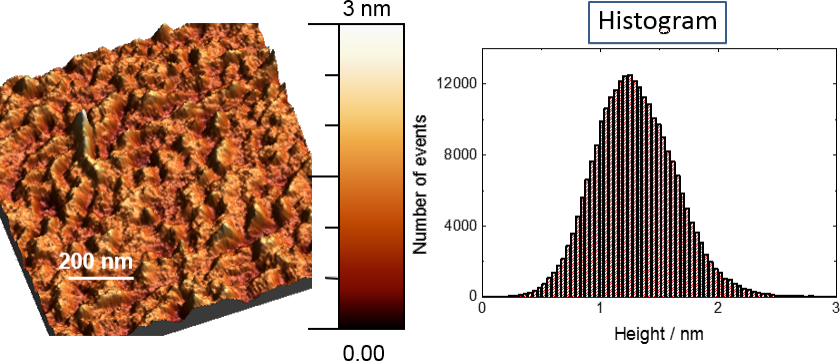
AFM topography characteristics considering a 1 × 1 μm^2^ area, after deposition of [Ni_2_L(HL^5^)](ClO_4_) (**7**) on gold with an rms roughness of 13 Å.

**Figure 6:**
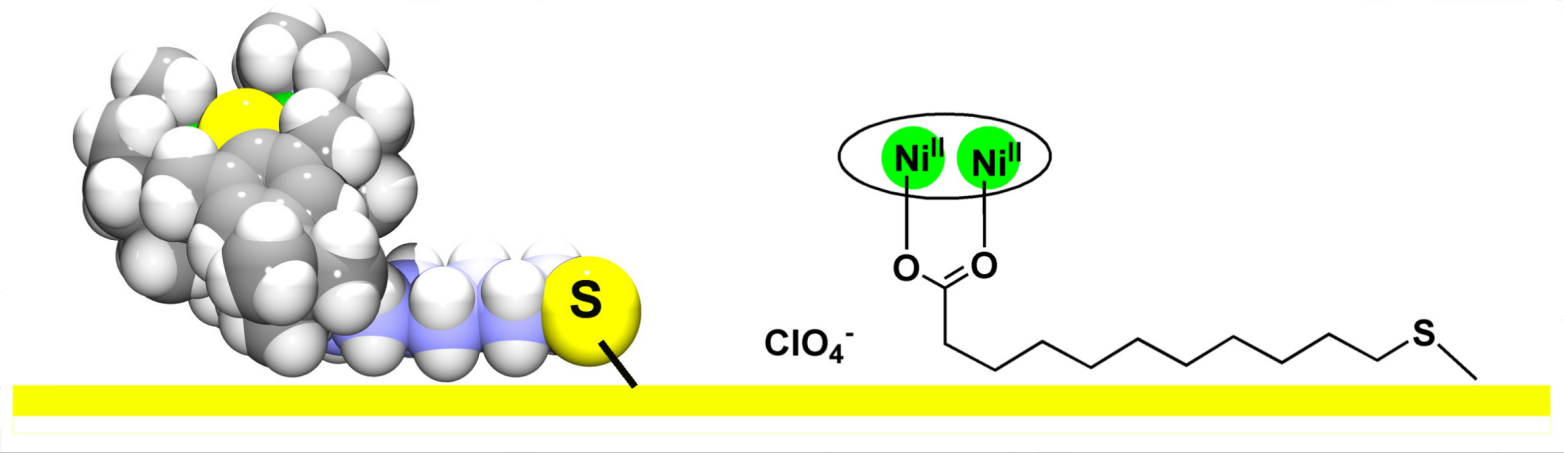
Proposed binding mode of complex **7** to gold (left: van der Waals representation of the [Ni_2_L(L^5^)]^+^ cation; right: representation with a Lewis formula. The macrocycle is shown as an ellipse encircling the two Ni^2+^ ions for clarity.

The topography of the gold surfaces was further investigated by AFM microscopy. Figure 5 shows the topography of a sample of [Ni_2_L(HL^5^)](ClO_4_) (**7**) on Au, which is representative. The topographic analysis of the bare gold and of complexes **2** and **8** are supplied in Supporting Information File 1. The presence of adsorbed complexes is indicated by the larger rms roughnesses of the samples (**7**: 13 Å, **8**: 19 Å) relative to that of the bare gold (6 Å). Similar rms roughnesses were previously found for **2** (17 Å) and **3** (17 Å), and the values match quite well with the estimated diameter of 12 Å of the globular-shaped [Ni_2_L(L’)]^+^ cation (neglecting the ClO_4_^−^ ion). We attribute the smaller rms roughness of **7** (13 Å) (relative to that of **8** (19 Å)) to be a consequence of the specific coordination mode of the coligand (see Figure 6).

For complexes **7** and **8** the film thickness was also investigated by spectroscopic ellipsometry. The ellipsometry data were fitted with the aid of the appropriate Cauchy model [60–61] as done for **2** and **3**. Others authors use *A* = 1.50, which in the case of the present monolayers would only make a thickness difference of about 0.1 nm. Reasonable fits were produced resulting in average thicknesses of 20 ± 7 Å for **7** and 24 ± 8 Å for **8**, which agree reasonably well with those determined by AFM. The thickness for **7** is again found to be smaller than that of **8**, indicative of an effect of the length of coligand on the thickness of the layer. Overall, the contact angle, AFM and ellipsometric data support the assumption that all complexes form monolayers rather than multilayer films, with the cationic [Ni_2_L(L’)]^+^ molecules covalently bound to the Au surface.

### Surface binding model

On the basis of the crystal structure of complex **7** and the AFM and ellipsometry measurements above, one can propose a specific orientation on the gold surface, as sketched in Figure 6. Note that the coligands in the complexes **2**, **3**, and **8** are more or less buried in the binding pocket of the [Ni_2_L]^2+^ fragment, and so the film thicknesses are determined largely by the dimensions of the [Ni_2_L]^2+^ dication alone. In case of complex **7**, however, approximately half of the coligand protrudes out of the pocket, and a coplanar orientation of this part of the coligand to the Au surface is likely. The smaller film thickness of **7** would be consistent with this in view of the proposed absorption model. Also, in the absence of packing and self-assembly of hydrocarbon chains, a coplanar binding of alkane thiol to gold is possible [62]. It is likely that van der Waals interactions exist between the Au surface and the alkyl (methylene and methyl) groups of the supporting ligands.

### Transport measurements

In orienting experiments, the deposition of complex **2** on gold was further investigated by transport measurements using an approach based on self-rolled-up nanomembranes [63–64]. Standard two point measurements at room temperature were carried out for the electrical characterization of the heterojunctions (Supporting Information File 1). The data analysis provides a characteristic transition voltage (*V*_T_) of 0.50 ± 0.05 V, a value which, albeit rather indicative than definitive, is in good agreement with the value of ≈0.6 V reported for monolayers of conjugated thiols on gold (≈0.6 V) [65]. According to these data, layers of paramagnetic complexes of **2** on gold behave as ultrathin insulating tunnel barriers, comparable to the self-assembled alkanethiol monolayers reported in previous work.

## Conclusion

In conclusion, three new mixed ligand complexes bearing ω-mercapto-carboxylato ligands were designed with the aim of facilitating the chemisorption of large magnetic bi-metallic complexes on gold surfaces by self-assembling of monolayers. The synthesis of [Ni_2_L(O_2_C(CH_2_)_5_SH)](ClO_4_) (**6**), [Ni_2_L(O_2_C(CH_2_)_10_SH)](ClO_4_) (**7**), and [Ni_2_L(O_2_C(C_6_H_4_)_2_SH)](ClO_4_) (**8**) was successfully realized. The coligands act invariably in a bridging mode as established by IR and UV–vis spectroscopy, ESIMS, and X-ray crystallography (for **6** and **7**). The structures of the complexes bearing ω-thiolalkane-carboxylates (H_2_L^4^, H_2_L^5^) are distinguished from the aromatic variants (H_2_L^2^, H_2_L^6^) by a bent conformation of the alkyl chain (alkyl chain perpendicular to the O_2_CC–C bond), such that the surface active thiol groups protrude laterally out of the cleft-like binding pocket of the [Ni_2_L]^2+^ fragments. The reactivity of complexes **6**–**8** is reminiscent of that of pure thiolato ligands. All are readily chemisorbed on Au surfaces as ascertained by contact angle measurements, AFM, spectroscopic ellipsometry and preliminary transport measurements (for **2**). The results may suggest that the length of the coligand affects the thickness of the corresponding films.

## Experimental

### Synthesis of compounds

All manipulations were carried out using standard Schlenk techniques and vacuum-line manipulations under a protective atmosphere of nitrogen unless otherwise stated. Compound **1** was prepared according to the published procedure [43]. All other compounds were purchased from commercial sources unless otherwise specified. The solvents were distilled prior to use and were deaerated according to standard procedures [66]. The IR spectra were recorded as KBr disks using a Bruker Tensor 27 FTIR spectrophotometer. UV–vis spectra were recorded on a Jasco V-670 UV/vis/near-IR spectrophotometer. Elemental analyses were carried out on a Vario EL elemental analyzer. NMR spectra were recorded on a Bruker AVANCE DPX-200 spectrometer at 300 K. ^1^H and ^13^C chemical shifts refer to solvent signals. ESIMS spectra were recorded on a Bruker Daltronics ESQUIRE3000 PLUS spectrometer. Temperature-dependent magnetic susceptibility measurements on powdered solid samples were carried out using a MPMS 7XL SQUID magnetometer (Quantum Design) over a temperature range 2–330 K at an applied magnetic field of 0.1, 0.5, and 1.0 Tesla. The observed susceptibility data were corrected for underlying diamagnetism.

**Safety note!** Perchlorate salts of transition metal complexes are hazardous and may explode. Only small quantities should be prepared and great care should be taken.

#### Synthesis of H_2_L^6^, step 1, 4-methylphenylboronic acid

Magnesium turnings (1.70 g, 70.2 mmol) were placed in a nitrogen-flushed three-neck vessel equipped with a dropping funnel and a reflux condenser. 40 mL of THF and a small grain of iodine were added to the vessel. A solution of 4-bromotoluene (7.19 mL, 10.0 g, 58.46 mmol) in 20 mL of THF was added drop wise to the solution to keep the solvent refluxing. The reaction mixture was kept under reflux for another 8 h. The resulting suspension was cooled to −80 °C and a solution of B(OMe)_3_ (7.3 mL, 6.80 g, 65.84 mmol) in 20 mL of THF was added drop wise. After complete addition, the mixture was allowed to warm up to room temperature. 35 mL of concentrated aqueous HCl (10%) were added for hydrolysis of unreacted B(OMe)_3_. The aqueous phase was separated and extracted with ether (4 × 15 mL). The organic phase was washed with brine, dried over MgSO_4_ and evaporated to dryness. The residue was recrystallized from an ethanol/water (1:1) solvent system to give 4-methylphenylboronic acid as colorless needles (2.94 g, 21.63 mmol, 37%). ^1^H NMR (400 MHz, CDCl_3_) δ 2.42 (s, 3H, CH_3_), 7.29 (d, ^3^*J* = 7.5 Hz, 2H, ArH), 8.13 (d, ^3^*J* = 7.5 Hz, 2H, ArH). This material was used without further purification in the next step.

#### Synthesis of H_2_L^6^, step 2, 4-carboxyphenylboronic acid

To a solution of KMnO_4_ (5.71 g, 36.13 mmol) and Bu_4_NBr (0.17 g, 0.53 mmol) in water (175 mL) a solution of 4-methylphenylboronic acid in 10% aqueous NaOH (20 mL) was added. The reaction mixture was stirred at 60 °C for 5 h. EtOH (8 mL) was added and the suspension was stirred for an additional 10 min to destroy the remaining KMnO_4_. The mixture was filtered and reduced to 1/2 of its original volume. The pH was adjusted to 1–2 using 2 M aqueous HCl. The resulting white precipitate was collected and dried in vacuo to give 4-boronobenzoic acid as a white powder (2.07 g, 12.47 mmol, 35%). ^1^H NMR (300 MHz, acetone-*d*_6_) δ 7.96–8.03 (m, 4H, ArH). This material was used without further purification in the next step.

#### Synthesis of H_2_L^6^, step 3,4'-(methylthio)-[1,1'-biphenyl]-4-carboxylic acid

4-Boronobenzoic acid (3.00 g, 18.08 mmol), 4-bromothioanisole (4.41 g, 21.70 mmol), Pd(OAc)_2_ (20.2 mg, 0.09 mmol) and K_2_CO_3_ (8.75 g, 63.28 mmol) were suspended in a PEG 400/H_2_O 1:1 solvent system (110 mL). The reaction mixture was stirred for 6 days at 60–65 °C, cooled down to room temperature and filtered. The filter cake was extracted repeatedly with diethyl ether. The ether phase was removed under reduced pressure and the pH of the remaining aqueous solution adjusted to 1 using 2 M aqueous HCl. The precipitate was filtered, washed with a small amount of dichloromethane to give the crude material, which was redissolved in acetone and filtered. Evaporation of the solvent gave 4'-(methylthio)-[1,1'-biphenyl]-4-carboxylic acid as a white solid (1.15 g, 4.71 mmol, 26% based on 4-boronobenzoic acid). ^1^H NMR (300 MHz, DMSO-*d*_6_) δ 2.53 (s, 3H, CH_3_), 7.38 (d, *^3^J* = 9 Hz, 2H, ArH), 7.71 (d, *^3^J* = 9 Hz, 2H, ArH), 7.71 (d, *^3^J* = 9 Hz, 2H, ArH). This material was used without further purification in the next step.

#### Synthesis of H_2_L^6^, step 4,4'-mercapto-[1,1'-biphenyl]-4-carboxylic acid

4'-(Methylthio)-[1,1'-biphenyl]-4-carboxylic acid (428 mg, 1.75 mmol) and NaSCH_3_ were dissolved in degassed *N*-methyl-2-pyrrolidone (40 mL) and stirred at 110 °C for 72 h. The solvent was removed in vacuo, the resulting residue was suspended in degassed 20% aqueous HCl (50 mL) and the mixture was stirred at room temperature for 30 min and at 40 °C for an additional 45 min. The solid was filtered off, washed with water (3 × 75 mL) and dried under vacuum. Yield: 387 mg (1.68 mmol, 96%), white air-sensitive powder. This material was used without further purification in the next step. ^1^H NMR (300 MHz, DMF-*d*_7_) δ 4.00 (s, 1H, SH), 7.40–8.10 (m, 8H, 8 ArH); ^13^C NMR (75 MHz, DMF-*d*_7_) δ 127.02 (*C*-COOH), 128.28 (*C*-HS), 130.46 (C1’), 162,24, 162,63, 162,83 (C4), 163,03, 167,64 (COOH); ESI^+^-MS (MeCN) *m*/*z*: 231,05 [M + H]^+^; IR (KBr pellet) 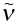: 2985 (m), 2850 (m), 2671 (m), 2550 (m, ν(SH)), 1684 (s, ν(RCO_2_)), 1607(s, ν(RCO_2_)), 1575 (m), 1521 (w), 1483 (m), 1425 (s), 1396 (m), 1297 (s), 1283 (s), 1199 (w), 1179 (w), 1130 (w), 1106 (m), 1017 (w), 1004 (w), 942 (w), 865 (w), 824 (s), 771 (s), 753 (w), 697 (w), 679 (w), 556 (w), 542 (w), 482 (w) cm^−1^

#### Preparation of [Ni_2_L(µ-O_2_C(CH_2_)_5_SH)]ClO_4_ (**6**)

This compound was prepared from **1** and 6-mercaptohexanoic acid by the procedure detailed for **2**. Yield: 191.1 mg (0.186 mmol, 56%). Recrystallization from a MeOH/EtOH 1:1 solvent system provided the title compound as a green microcrystalline solid, which was washed with EtOH and ether and dried in vacuum. Yield: 191.1 mg (0.186 mmol, 56%). ESI^+^-MS (MeCN) *m*/*z*: 932.39 [M^+^]; IR (KBr pellet) 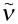: 2962 (s), 2902 (s), 2868 (s), 2808 (m), 2547 (w, ν(SH)), 1717 (w), 1596 (s, ν_as_(RCO_2_)), 1565 (m), 1546 (w), 1461 (s), 1407 (s, ν_s_(RCO_2_)), 1363 (m), 1309 (w), 1292 (w), 1264 (w), 1233 (w), 1152 (m), 1096 (s), 1060 (s), 1039 (s), 846 (m), 824 (s), 752 (s), 623 (s) cm^–1^; UV–vis (CH_3_CN) λ_max_/nm (ε/M^−1^ cm^−1^): 209 (40040), 270 (15840), 304 (14100), 327 (11900), 372 (2150), 451 (140), 649 (26), 1129 (63); anal. calcd for C_44_H_75_ClN_6_Ni_2_O_6_S_3_ (1033.14): C, 51.15; H, 7.32; N, 8.13; found: C, 50.78; H, 7.26; N, 8.05. This compound was additionally characterized by X-ray crystallography.

#### Preparation of [Ni_2_L(µ-O_2_C(CH_2_)_10_SH)]ClO_4_ (**7**)

A solution of **1** (200 mg, 0.217 mmol), 11-mercaptoundecanoic acid (62 mg, 0.285 mmol), and NEt_3_ (28 mg, 40 µL, 0.282 mmol) was stirred at room temperature for 8 h. A solution of LiClO_4_·3H_2_O (348 mg, 2.17 mmol) in degassed EtOH (15 mL) was added. The solution was reduced to about 10 mL. The resulting pale-green precipitate was filtered off, washed with EtOH and ether and dried in vacuum. Recrystallization from a MeOH/EtOH 1:1 solvent system provided the title compound as a green, microcrystalline solid, which was washed with EtOH and ether and dried in vacuum. Yield: 158 mg (0.143 mmol, 66%). ESI^+^-MS (MeCN) *m*/*z*: 1002.5 [M^+^]; IR (KBr pellet) 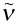: 3600–3300 (m), 2962 (s), 2902 (s), 2868 (s), 2808 (m), 2547 (w, ν(SH)), 1717 (w), 1581 (s, ν_as_(RCO_2_)), 1565 (m), 1546 (w), 1461 (s), 1423 (s, ν_s_(RCO_2_)), 1363 (m), 1309 (w), 1292 (w), 1264 (w), 1233 (w), 1152 (m), 1096 (s), 1060 (s), 1039 (s), 846 (m), 824 (s), 752 (s), 623 (s) cm^−1^; UV–vis (CH_3_CN) λ_max_/nm (ε/M^−1^ cm^−1^): 202 (91450), 279 (28200), 331 (14140), 373 (2200), 453 (142), 650 (30), 1133 (64) nm; anal. calcd for C_51_H_73_ClN_6_Ni_2_O_6_S_3_·3H_2_O (1115.20 + 54.03): C, 52.39; H, 6.81; N, 7.19; found: C, 52.77; H, 6.51; N, 7.23.

#### Preparation of [Ni_2_L(µ-O_2_C(C_6_H_4_)_2_SH)]ClO_4_ (**8**)

The dinuclear nickel complex **1** (307 mg, 0.333 mmol) and 4'-mercapto-[1,1'-biphenyl]-4-carboxylic acid (76.6 mg, 0.333 mmol) were dissolved in a nitrogen-purged MeOH/CH_2_Cl_2_ (1:1) solvent mixture (50 mL). NEt_3_ (34 mg, 46 µL, 0.333 mmol) was added and the resulting green solution was stirred at room temperature for 8 hours. A solution of LiClO_4_·3H_2_O (534.2 mg, 3.33 mmol) in degassed EtOH (20 mL) was added. The solution was reduced to about 10 mL. The resulting pale-green precipitate was filtered off, washed with EtOH and ether and dried in vacuum. Recrystallization from a EtOH/MeCN 1:1 solvent system provided the title compound as a green, microcrystalline solid, which was washed with EtOH and ether and dried in vacuum. Yield: 219.1 mg (0.196 mmol, 59%). ESI^+^-MS (MeCN) *m*/*z*: 1013.37 [M^+^]; IR (KBr pellet) 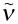: 3600–3300 (m), 2962 (s), 2902 (s), 2868 (s), 2808 (m), 2548 (w, ν(SH)), 1717 (w), 1596 (s, ν_as_(RCO_2_)), 1565 (m), 1546 (w), 1461 (s), 1408 (s, ν_s_(RCO_2_)), 1363 (m), 1309 (w), 1292 (w), 1264 (w), 1233 (w), 1152 (m), 1096 (s), 1060 (s), 1039 (s), 846 (m), 824 (s), 752 (s), 623 (s) cm^−1^; UV–vis (CH_3_CN) λ_max_/nm (ε/M^−1^ cm^−1^): 202 (91450), 279 (28200), 331 (14140), 373 (2200), 453 (142), 650 (30), 1118 (64); anal. calcd. for C_51_H_73_ClN_6_Ni_2_O_6_S_3_·3H_2_O (1115.20 + 54.03): C, 52.39; H, 6.81; N, 7.19, found: C, 52.77; H, 6.51; N, 7.23.

### X-ray crystallography

Crystals of **6** and **7** were grown by slow evaporation of a mixed acetonitrile/ethanol solvent system and subjected to diffraction experiments on a STOE-IPDS-2T-diffractometer. Graphite-monochromated Mo Kα radiation (λ = 0.71073 Å) was used throughout. The data were processed with X-AREA and corrected for absorption using STOE X-Red32 [67]. The structures were solved by direct methods (SHELXS-2013) [68] and refined by full-matrix least-squares on *F*^2^. However, the quality of the refinement for the two compounds was very low. The ClO_4_^−^ anions and solvate molecules could not be located, and so the structures can only serve to validate the atom connectivity of the complex cations.

#### Crystal data for [Ni_2_L(O_2_C(CH_2_)_5_SH)](ClO_4_) (**6**)

C_44_H_75_ClN_6_Ni_2_O_6_S_3_, *M*_r_ = 1033.14 g/mol, triclinic, space group *P*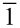, *a* = 13.6528(9) Å, *b* = 13.9237(9) Å, *c* = 16.2949(10) Å, α = 69.323(5), β = 74.211(5)°, γ = 86.429(5)°, *V* = 2786.8(3) Å^3^, *Z* = 2, ρ_calcd_ = 1.113 g/cm^3^, *T* = 180 K, μ(Mo Kα) = 0.823 mm^–1^ (λ = 0.71073 Å), 23125 reflections measured, 10911 unique, 8853 with *I* > 2σ(*I*). Final R1 = 0.0784, wR2 = 0.2512 (*I* >2σ(*I*)). The ClO_4_^–^ anion could not be located. The structure contains large (solvent accessible voids) of ≈750 Å^3^ attributed to MeCN or EtOH solvate molecules. Only the structure of the complex cation could be identified.

#### Crystal data for [Ni_2_L(O_2_C(CH_2_)_10_SH)](ClO_4_) (**7**)

C_49_H_85_ClN_6_Ni_2_O_6_S_3_, *M*_r_ = 1103.27 g/mol, monoclinic, space group *P*2_1_/*n*, *a* = 21.689(4) Å, *b* = 13.593(3) Å, *c* = 21.698(4) Å, β = 104.20(3)°, *V* = 6201(3) Å^3^, *Z* = 4, ρ_calcd_ = 1.182 g/cm^3^, *T* = 180 K, μ(Mo Kα) = 0.80 mm^–1^ (λ = 0.71073 Å), 34319 reflections measured, 11365 unique, 4520 with *I* > 2σ(*I*). Final R1 = 0.1247, wR2 = 0.3743 (*I* >2σ(*I*)). The ClO_4_^–^ anion could not be located. The structure contains large (solvent accessible voids) of ≈1000 Å^3^ attributed to MeCN or EtOH solvate molecules. Only the structure of the complex cation could be identified.

### Computational details

DFT calculations were carried out utilizing density functional theory (DFT). Perdew, Burke and Ernzerhof’s PBE0 hybrid functional [69–70] and Ahlrich’s triple-zeta valence basis set (TZV(P)) [71] were used. Calculations were performed with the ORCA [72–73] program package (revision 3.0.3) as previously described [59]. The coordinates were taken from the crystal structures and were fixed during the calculations.

### Contact angle measurements

Surface hydrophobicity was examined by performing water contact angle measurements with a DSA II (Krüss, Hamburg, Germany) contact angle analyzer. The contact angle measurements were collected using a 4 μL drop size of deionized, distilled water. At least 5 contact angles per five different locations were averaged.

### Atomic force microscopy

An Agilent 5600LS AFM system was used to collect topography data under Ar and ambient conditions in order to keep the integrity of the organic system. Measurements were performed in tapping mode in order to minimize the contact between the AFM probe and the sample surface and avoid damage or modification of the topographic characteristics. Special ultrasharp (4–10 nm tip radius) Olympus cantilevers were employed, allowing high sensitivity measurements. Data shown in the respective Figures correspond to a 1 × 1 μm^2^ area, although a mapping of the topographic characteristics was performed on different points of the samples in order to verify the uniformity of the organic system over the Au substrate.

### Ellipsometry

Spectroscopic ellipsometric measurements were conducted on a J. A. Woollam Co., Inc. M-2000 T-Solar ellipsometer operating with a xenon lamp. Ellipsometry scans were recorded under ambient conditions in a spectral range from 0.7 eV to 5 eV at light incidence angles of 65°, 70°, and 75°. The samples were immediately measured after preparation. Gold substrates were cleaned, immersed in a 1 × 10^−3^ M solution of the complexes in CH_2_Cl_2_ for at least 12 h, rinsed with absolute ethanol and dried in a stream of ultrahigh purity nitrogen. The modelling environments CompleteEASE and WVASE32 (both J. A. Woollam Co., Inc.) were used for data evaluation. The dielectric function of a pristine gold substrate, measured and modelled in agreement with database values, was taken as a reference substrate layer for all measurements. For each molecular complex, ellipsometry spectra were recorded for at least nine different locations on the sample surface. In a parameter-coupled fitting procedure, the organic film thickness values were determined using a Cauchy dispersion model that is commonly applied for the refractive index of monolayers described as transparent media. Error bars represent the standard deviation from the mean thickness value within one sample series. The scatter observed in the data was typically ±0.6 nm, arising most likely from a film roughness of the gold substrates of about 0.6 nm (measured by AFM).

### Transport measurements

Devices used for transport measurements were fabricated on silicon substrates employing standard photolithographic processes combined with thermal deposition. The top electrode was prepared by rolling a metallic nanomembrane over a monolayer of chemisorbed molecules of **2** previously synthesized on a thin gold film deposited onto a silicon pillar (for details, see Supporting Information File 1).

## Supporting Information

File 1 Additional AFM topography images, detailed description of transport measurements.
