# Supplementary material for: Deposition of exchange-coupled dinickel complexes on gold substrates utilizing ambidentate mercapto-carboxylato ligands
Source: Beilstein J Nanotechnol. 2017 Jul 5;8:1375–87. doi: 10.3762/bjnano.8.139 (PMC5530607; doi:10.3762/bjnano.8.139)
Supplement: File 1 — Additional AFM topography images, detailed description of transport measurements. [file Beilstein_J_Nanotechnol-08-1375-s001.pdf]

Deposition of exchange-coupled dinickel complexes on gold substrates utilizing ambidentate mercapto-carboxylato ligands

Martin Börner[1], Laura Blömer[1], Marcus Kischel[1], Peter Richter[2], Georgeta Salvan[2], Dietrich R. T. Zahn[2], Pablo F. Siles[3,4], Maria E. N. Fuentes[4], Carlos C. B. Bufon[4], Daniel Grimm[4], Oliver G. Schmidt[4], Daniel Breite[5], Bernd Abel[5], and Berthold Kersting*[1]

Address: [1]Institut für Anorganische Chemie, Universität Leipzig, Johannisallee 29, 04103 Leipzig, Germany, [2]Semiconductor Physics, Chemnitz University of Technology, D-09107 Chemnitz, Germany, [3]Material Systems for Nanoelectronics, Chemnitz University of Technology, Reichenhainer Str. 70, 09107 Chemnitz, Germany, [4]Institute for Integrative Nanosciences, IFW Dresden, Helmholtz Str. 20, 01069 Dresden, Germany, and [5]Leibniz-Institute of Surface Modification (IOM), Permoser Str. 15, D-04318 Leipzig, Germany

* Corresponding author

Email: Berthold Kersting - b.kersting@uni-leipzig.de

Dedicated to Prof. Dr. Dr. Evamarie Hey-Hawkins on the occasion of her 60th birthday.

Additional Experimental Information

Content:

1) AFM topography image of complex **2** on Au.

2) AFM topography image of Au substrate used for deposition of **7** and **8**.

3) AFM topography image of complex **8** on Au.

4) Transport measurements.

1. AFM topography image of Complex 2 on Au.

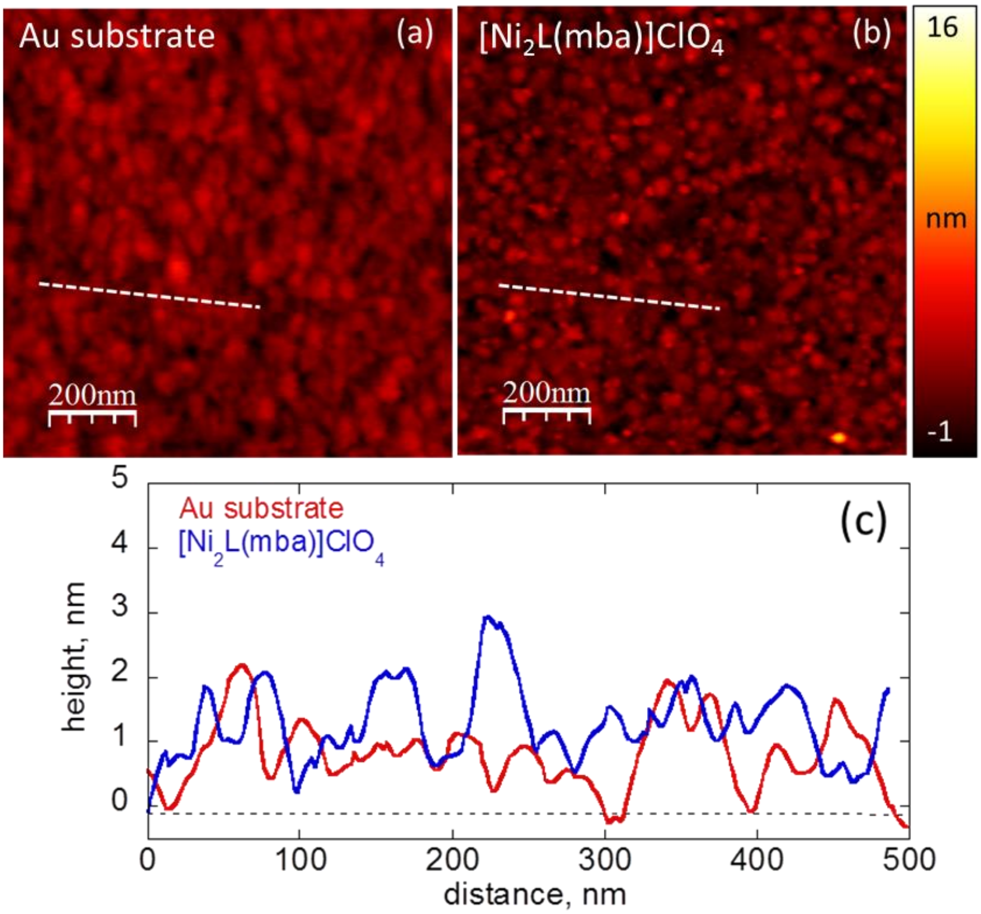

Figure S1. AFM topography characteristics of a 1×1 μm^2 area, (a) Au substrate *as grown* with a roughness of 0.6 (1) nm (rms), (b) Au substrate after deposition of **2** with a roughness of 0.9 (1) nm (rms), (c) AFM profiles following the white-dotted line

in (a) and (b). Substrates were prepared by magnetron sputtering of 100 nm Au onto polished silicon wafers Si(111), pre-coated with a 20 nm thick adhesion layer of titanium. The substrates were cut into slides (1 cm × 1 cm) and cleaned by ultrasonic treatment in piranha solution (H_2SO_4/H_2O_2: 3/1) followed by washing with dichloromethane, ethanol, and deionized water.

2. AFM topography image of Au substrate used for deposition of 7 and 8.

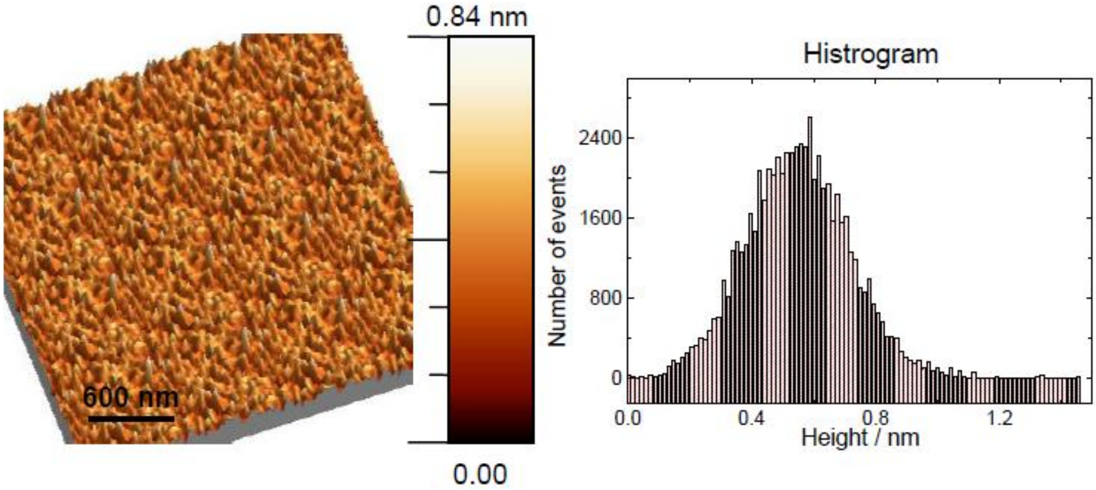

Figure S2. AFM topography characteristics of the Au substrate (used for deposition of complexes **7** and **8**) with a roughness of 0.6 (1) nm (rms).

3. AFM topography image of complex 8 on Au.

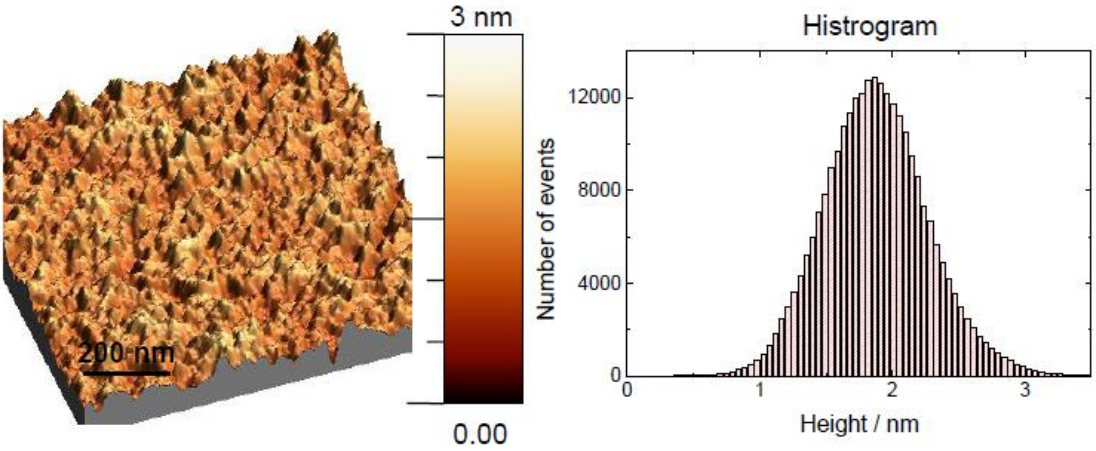

Figure S3. AFM topography characteristics of a 1×1 μm^2 area of the Au substrate after deposition of complex **8**.

4. Transport measurements. Devices used for transport measurements were fabricated on silicon substrates employing standard photolithographic processes combined with thermal deposition. The top electrode is prepared by rolling a metallic nanomembrane over a monolayer of chemisorbed molecules of **2** previously synthesized on a thin gold film deposited onto a silicon pillar (see Figure S4a,c).

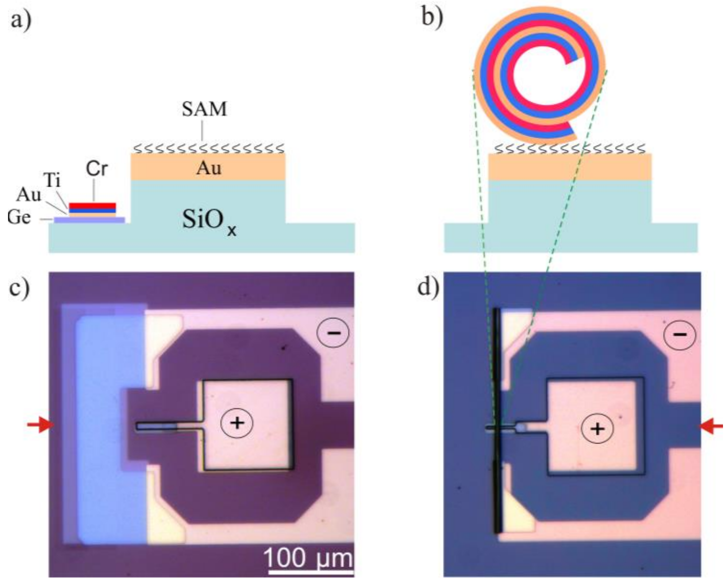

Figure S4. Schematics and optical images of the molecular thin film device. a) The unrolled device consists of a strained, detachable metal film (left) and a SiO_2 pillar with a deposited thin gold film as well as monolayer of **2** (bottom electrode, right). c) The two electrodes, denoted + and –, are disconnected. b) After releasing and self-curling of the strained layers, the tubular top electrode (-) contacts the SAM (here layers of chemisorbed **2** and a new current path is deterministically created across the molecular layer. d) Both electrodes are connected and the contact area is restricted to the pillar region. The red arrows indicate the horizontal plane of the cross-sectional schematic drawings. Here, the vertical dimensions are exaggerated for more clarity and the different layers are not in scale.

These pillars form the bottom contact of the hybrid devices. The monolayer is grown on the gold pillar by introducing the samples in a 1mM solution of the complexes in dichloromethane. The strained metallic nanomembrane used as top electrode is made by the sequential deposition of Au, Ti and Cr thin films on a GeO_x sacrificial layer. In order to establish the electrical contact with the monolayer, the strained

metallic nanomembrane is selectively released from the substrate by removing the GeO_x sacrificial layer in water. A layer of **2** remains chemisorbed under these conditions, as shown by contact angle measurements. The strained metallic layers relax upon release, which results in an up-rolling of the tubular top electrode, contacting the molecular layer in a soft manner (Figure S4 b,d). A new current path is generated from the top electrode across the SAM to the bottom electrode.

Standard two point measurements at room temperature were carried out for the electrical characterization of the heterojunctions. Figure S5a shows a typical measurement plot according to the Fowler-Nordheim equation (Eq. S1):

$$I \propto V^2 \exp\left(-\frac{4d\sqrt{2m_e\phi^3}}{3\hbar qV}\right) \qquad \text{(S1)}$$

where d is the barrier width, m_e is the electron effective mass, ϕ is the barrier height, and q is the electronic charge. Using this equation, we can represent the measurement data using the expression in relation S2.

$$\ln\frac{I}{V^2} \sim \frac{1}{V} \qquad \text{(S2)}$$

The minimum value observed in Figure S5a indicates the point where a transition from direct tunneling to field emission takes place.[1] In previous work where alkanethiols were connected by metallic electrodes, such a transition has been interpreted as the barrier shape modification. In this particular case, the tunneling barrier height is the difference between the Fermi level of the metal and the molecular HOMO/LUMO level. For applied voltages lower than the metal/molecule barrier height, the barrier shape is maintained trapezoidal and the main transport mechanism is direct tunneling. In this case, as a first approximation of the Simons model,[1] the dependence of the current with the voltage is typically linear. As the

voltage is increased, the molecular barrier becomes field dependent, having a transition to a triangular shape. As shown in the Fowler-Nordheim plot, the transition voltage (V_T) of 0.50 ± 0.05 V obtained in this work is in good agreement with those reported for conjugated thiols groups (~0.6V).[1] The dispersion probably comes from samples with some inhomogeneities of the molecular layer or changes in the Au-SAMs bonding.

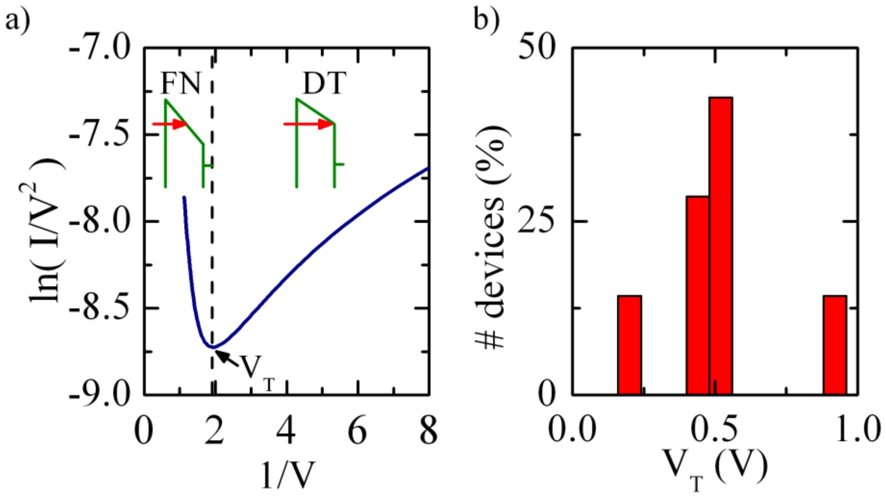

Figure S5. a) Representative Fowler-Nordheim plot with a clear transition at V_T from direct tunneling (DT) to Fowler-Nordheim emission (FN). The dashed line indicates the separation of the two regimes, with the respective barrier shape schematically shown. b) Transition voltage distribution found for the measured current-voltage characteristics with an error of ±0.1 V.

1. Beebe, J. M.; Kim, B.; Frisbie, C. D., Kushmerick, J. G. *ACS Nano,* **2008**, *2*, 827–832.
